# Untangling ccRCC prognosis with SLINKY

**DOI:** 10.18632/oncotarget.15536

**Published:** 2017-02-20

**Authors:** Bradley I. Reinfeld, W. Kimryn Rathmell

**Affiliations:** Department of Medicine, Division of Hematology and Oncology, Vanderbilt University Medical Center, Vanderbilt-Ingram Cancer Center, Nashville, TN, USA

**Keywords:** renal cell carcinoma, lincRNA, SLINKY, HNRNPK, prognostic

Renal Cell Carcinoma (RCC) is a heterogeneous set of diseases. Even within the major histological subtype of clear cell renal cell carcinoma (ccRCC), there is substantial variability in patient prognosis. The ultimate goal of organizing this heterogeneous disease is to elucidate prognostic elements and aid in clinical and therapeutic decision-making [1]. In organ-confined disease, there remains significant risk of recurrence, currently defined by combinations of clinical and pathological variables. It is the uncertainty of prognosis in this stage of disease that drives current biomarker development.

Currently, there are no clinically implemented biomarkers to predict which patients have a higher likelihood of disease recurrence. In an age of precision medicine, can a prognostic test be developed to aid clinicians in management of patients with early stage RCC? How do we decide which patients require yearly follow-up, which patients need to receive adjuvant therapy, or which patients need to enroll in a clinical trial to decrease their chance of recurrent metastatic disease? We have arrived in an era where adjuvant therapy is still reporting mixed results, but with one positive study, and many more studies to report in the near future [2], we will very soon be pressed to identify high risk patients with precision.

In ccRCC, previous work has attempted to uncover such prognostic factors. The Recurrence Score (RS) and ClearCode34 are two of many efforts to develop transcript signatures with prognostic significance. The RS is a 16 gene panel validated in large patient cohorts to provide more accurate prognostic guidance than clinical and pathological variables alone [3]. ClearCode34 divides ccRCC into two subtypes (ccA/ccB) based on a validated 34-gene expression panel. ccB patients have significantly decreased overall survival, and this stratification showed superior performance to clinical algorithms alone [6]. However, these and other markers are hindered by issues related to the high degree of heterogeneity in ccRCC [4]. Thus, recent studies have turned to functional imaging as a potentially more holistic evaluation [7]. Additionally, specific tumor mutations have been found to correlate to overall patient survival. Patients expressing wildtype BAP1 and PBRM1 have longer median survival than patients who harbor mutation in one or both of these genes [8].

In this month’s Oncotarget, [5] describes a novel biomarker for stratification of prognosis in ccRCC. By re-annotating the ccRCC RNA-seq data from The Cancer Genome Atlas (TCGA), expression of previously overlooked long non-coding RNAs (lincRNA) were found to correlate to patient survival. These correlated lincRNAs were evaluated to an independent set of ccRCC patients from the University of Tokyo. Within this new cohort, increased expression of one validated lincRNA, Survival-predictive LincRNA in Kidney cancer (SLINKY), was found to independently predict worse prognosis. Further, ROC analysis of these Japanese patients illustrated that adding SLINKY expression predicts overall survival with more accuracy than staging and grading alone for all patients with Stage I, II and III disease.

Next, in vitro analysis characterized the impact of SLINKY on phenotypes of ccRCC cell lines. Knocking down SLINKY by siRNA resulted in significantly less cell proliferation, but no change in migration. BrdU labeling experiments illustrated that SLINKY-knockdown leads to cell cycle arrest during the G1 phase. Further, transcriptomic analysis uncovered a group of 93 genes whose expression significantly changed with SLINKY knockdown. This set of 93 genes was then re-applied to the TCGA dataset and found to correlate with SLINKY expression as well as predict overall patient survival, illustrating that the genetic program dictated by SLINKY in vitro is similar to the changes initiated by SLINKY in patients.

To elucidate SLINKY’s role, the Stanford group searched for the binding partner for this lincRNA. Protein microarray studies and RNA immunoprecipitation revealed that HNRNPK bound tightly to SLINKY. Furthermore, knocking down HNRNPK recapitulated the proliferation defects as well as global transcriptional changes as the siRNA knockdowns of SLINKY.

Ultimately, this work suggests that SLINKY- HNRNPK complex might repress a tumor suppressor, and that low levels of intact SLINKY- HNRNPK results in appropriate transcription of the growth-arresting signal. In aggressive tumors, overexpression of the SLINKY- HNRNPK complex represses the tumor suppression, leading to increased tumor growth. Regardless of SLINKY’s physiological role, the fact that SLINKY is specifically expressed in cancerous tissues and that increased SLINKY expression predicts decreased overall survival, makes it a worthy candidate as a prognostic ccRCC biomarker.

Single unit biomarkers provide the most simple and direct means of classifying disease. Further work will need to explore the heterogeneity of SLINKY expression across individual primary tumors. Interestingly, overexpression of SLINKY’s binding partner, HNRNPK, is known to be a negative predictor of survival in multiple cancer types (colorectal, bladder, head and neck squamous cell carcinoma, melanoma, hepatocellular carcinoma, breast, and esophageal). Now knowing a key binding partner for HNRNPK, future work may more accurately illustrate the role of this protein in tumorigenesis. Previous work argues that in myeloid malignancies HNRNPK is a haploinsufficient tumor suppressor while in solid tumors it functions an oncogene [9]. Teasing out the role of the SLINKY-HNRNPK complex may elucidate more signaling pathways that can be targeted to ultimately improve patient outcomes in patients with metastatic RCC, in addition to serving as a prognostic biomarker.
